# Factors Associated With State Mandate of Nursing Home Staff Testing During COVID-19

**DOI:** 10.1093/geroni/igab046.959

**Published:** 2021-12-17

**Authors:** Elizabeth Simpson, Edward Miller, Molly Wylie, Lisa Beauregard, Pamela Nadash, Michael Gusmano

**Affiliations:** 1 University of Massachusetts Boston, University of Massachusetts Boston, Massachusetts, United States; 2 University of Massachusetts Boston, Boston, Massachusetts, United States; 3 University of Massachusetts Boston, University of Massachusetts boston, Massachusetts, United States; 4 Executive Offce of Elder affairs, Executive Office of Elder Affairs, Massachusetts, United States; 5 Rutgers University School of Public Health, Rutgers University School of Public Health, New Jersey, United States

## Abstract

COVID-19 has presented challenges for nursing homes (NHs) and other congregate living settings which serve older adults at high risk for morbidity and death from the virus. This study identified factors associated with states’ adopting a mandate for regular staff testing for COVID-19 in NHs. Potential correlates included state government ideology and capacity, NH supply and demand, prevailing economic conditions, existing state policies, and NH characteristics. Findings indicate that percent for profit NHs is most strongly associated with adoption of a state staff testing mandate. Governing capacity (average legislative salary), percent population at risk for COVID-19, and existing public policy (percent Medicaid spending devoted to long-term services and supports (LTSS) were also associated with the probability of adoption. Based on these results, states with more proprietary facilities and greater capacities for government action, investment in Medicaid LTSS, and at-risk populations were more likely to mandate regular staff testing in NHs.

